# Phylogenetic distribution of large-scale genome patchiness

**DOI:** 10.1186/1471-2148-8-107

**Published:** 2008-04-11

**Authors:** José L Oliver, Pedro Bernaola-Galván, Michael Hackenberg, Pedro Carpena

**Affiliations:** 1 Dpto de Genética, Facultad de Ciencias, Universidad de Granada, Spain; 2 Dpto de Física Aplicada II, Universidad de Málaga, Spain; 3 Bioinformatics Group, CIC bioGUNE, CIBER-HEPAD, Technology Park of Bizkaia, 48160 Derio, Bizkaia, Spain

## Abstract

**Background:**

The phylogenetic distribution of large-scale genome structure (i.e. mosaic compositional patchiness) has been explored mainly by analytical ultracentrifugation of bulk DNA. However, with the availability of large, good-quality chromosome sequences, and the recently developed computational methods to directly analyze patchiness on the genome sequence, an evolutionary comparative analysis can be carried out at the sequence level.

**Results:**

The local variations in the scaling exponent of the Detrended Fluctuation Analysis are used here to analyze large-scale genome structure and directly uncover the characteristic scales present in genome sequences. Furthermore, through shuffling experiments of selected genome regions, computationally-identified, isochore-like regions were identified as the biological source for the uncovered large-scale genome structure. The phylogenetic distribution of short- and large-scale patchiness was determined in the best-sequenced genome assemblies from eleven eukaryotic genomes: mammals (*Homo sapiens*, *Pan troglodytes*, *Mus musculus*, *Rattus norvegicus*, and *Canis familiaris*), birds (*Gallus gallus*), fishes (*Danio rerio*), invertebrates (*Drosophila melanogaster and Caenorhabditis elegans*), plants (*Arabidopsis thaliana*) and yeasts (*Saccharomyces cerevisiae*). We found large-scale patchiness of genome structure, associated with *in silico *determined, isochore-like regions, throughout this wide phylogenetic range.

**Conclusion:**

Large-scale genome structure is detected by directly analyzing DNA sequences in a wide range of eukaryotic chromosome sequences, from human to yeast. In all these genomes, large-scale patchiness can be associated with the isochore-like regions, as directly detected *in silico *at the sequence level.

## Background

As soon as genome sequences of sufficient length were available, three groups [1-3] independently described powerful methods (power spectra, analysis of fluctuations in DNA walks) to study large-scale genome structure at sequence level. The emerging view was the existence of long-range, power-law correlations, thus pointing to fractal (scale-invariant) structure in DNA sequences. However, such fractal structure, implying the existence of DNA segments of all sizes, directly clashes with the view of the genome as composed of long, homogeneous segments (isochores).

Isochores - long (>>300 kb), compositionally fairly homogeneous genome regions of different average GC levels were uncovered by analytical ultracentrifugation of bulk DNA [4-10]. The phylogenetic distribution of isochores was traditionally studied by centrifugation techniques [7-11], but the analysis of base composition at third codon position or the comparison of GC content between coding and non-coding sequences [12-17] has been also used.

The paradox between a fractal (scale-invariant) or an isochore structure for the genome has been recently solved in the human genome by the discovery that correlations can show deviations from the power-law behavior [18]. Interestingly, such deviations can be associated to isochore-like regions [19] -long-homogeneous genome regions computationally predicted by directly examining the genome sequence and sharing many compositional and biological features with true isochores [20-24].

In this way, the phylogenetic distribution of large-scale genome patchiness can be now explored by analyzing the deviations of power-law behavior in long-range correlations. The method of choice is Detrended Fluctuation Analysis or DFA; the deviations from the power-law can then be revealed by the variations in the local behavior of the scaling exponent α [18,19]. Here, we determined the variation of α at different scales in a wide phylogenetic range of genome sequences. Our analysis clearly distinguishes two characteristic length scales, the larger of which is demonstrated to be unambiguously associated with the isochore-like regions, as detected *in silico*. The phylogenetic distribution of such patterns leads to insights in understanding the evolution of genome compositional heterogeneity.

## Results

### Two characteristic length scales in human DNA

**Figure 1:**
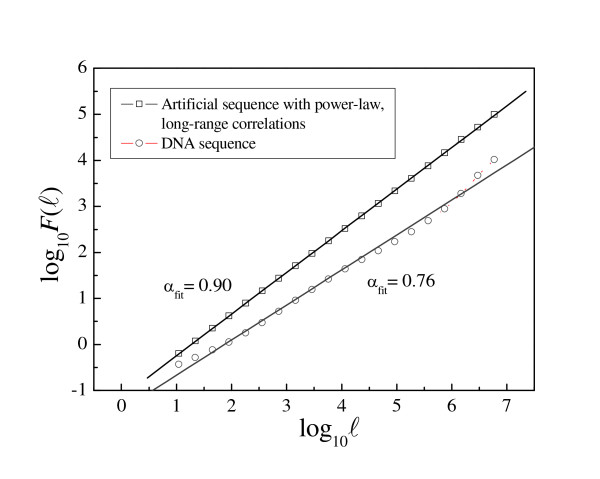
**Detecting long-range correlations through DFA**. Log-log plots of the fluctuation function F(ℓ) vs. the length scale ℓ. The scaling exponent α is given by the slope of the linear fit. The artificial sequence was generated using a standard method to create long-range correlated sequences [31] by imposing that α = 0.90, and the DFA recovers correctly this scaling exponent. The DNA sequence analyzed is the largest contig from human chr. IV mapped into a binary sequence using the SW (strong-weak) mapping rule: C or G → 1, A or T → 0. Its scaling exponent is α_fit _= 0.76.

**Figure 2:**
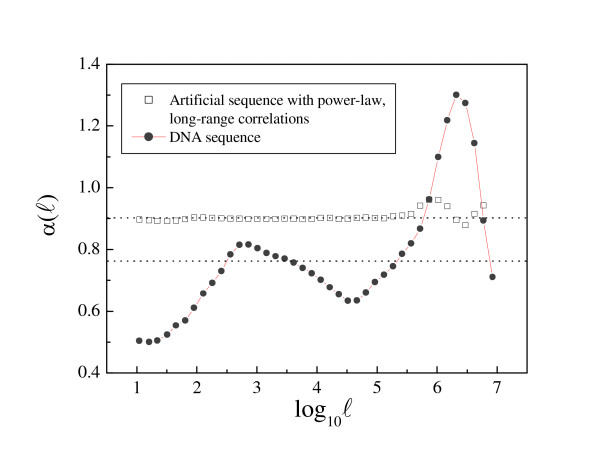
**Detecting deviations from power-law behavior**. The deviations from power-law behavior can be detected by plotting the local α value vs. the scale ℓ (see Methods). The sequence with real scaling presents a constant value of α(ℓ) with small fluctuations around the linear fitting value -α_fit _= 0.90 (dotted line). However, in human DNA, α(ℓ) is far from being a constant: the fluctuations around the value resulting from the linear fitting (α_fit _= 0.76, dotted line) are not only very large but also following a particular pattern, indicating that this α_fit _value is meaningless, and that there is no real scaling despite the good linear fitting.

Long-range correlations are detected by log-log plots of the fluctuation function F(ℓ) vs. the length scale ℓ. The scaling exponent α is then given by the slope of the linear fit (see Methods). Figures 1 and 2 show how α(ℓ) profiles are used to uncover characteristic scales in human DNA sequences. Significant deviations from the power-law behavior reveal two main characteristics scales in a large human sequence contig (chromosome IV, positions 75671304-167795054, 92.1 Mb). The intermediate scale goes from log_10 _ℓ > 1,5 (ℓ > 30 bp) to log_10 _ℓ ≈ 4.5 (ℓ ≈ 30 kb), while the large scale spans up to log_10 _ℓ ≈ 7 (ℓ ≈ 10 Mb). These values are representative for the entire human complement, but when the available contigs were shorter, the large scale typically extended only until log_10 _ℓ ≈ 6 (ℓ ≈ 1 Mb).

### Biological source for the two characteristic scales in human DNA

**Figure 3:**
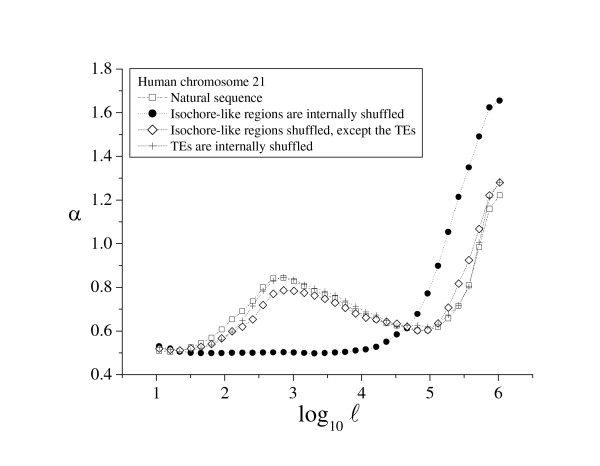
**α(ℓ) profile of the human chromosome 21**. Plot of the local α value vs. the scale ℓ – the α(ℓ) profile – in the human chromosome 21. The profiles for the natural sequence, the artificial sequence obtained after the *in silico *determined, isochore-like regions were internally shuffled, the artificial sequence obtained by shuffling the isochore-like regions without touching the TEs, and the artificial sequence obtained by shuffling the TEs are shown.

Short- and long-range genome structure is shaped by a wide range of structural, functional, and evolutionary factors acting on the DNA sequence: mutational bias, transcriptional activity, translational constraints, patterns of gene expression, open or closed state of the chromatin, insertion of repeat elements, etc. (see [7-10] for review). Because of its overlap, discerning the role of each of these factors is a difficult task. Experiments of selective shuffling of some genome regions, while leaving others intact, can be used to approach this problem. Here, we used such selective shuffling to identify the biological source for the two characteristic scales identified in DNA sequences [19]. As an example, Figure 3 shows the results for human chromosome 21. When the sequence segments corresponding to the isochore-like regions (as determined by the algorithm IsoFinder [24]) were internally shuffled, the small scale properties disappeared, and the α(ℓ) profile remained flat with a constant value of 0.5, as expected in a random sequence without compositional structure. The only effect of the shuffling in the large scale was a slight increase in the corresponding peak: as the shuffling homogenizes the small scale, the large scale patchiness becomes more discernible. Therefore, the large scale properties observed in the α(ℓ) profile can be unambiguously attributed to the computationally predicted, isochore-like regions [19]. However, when the isochore-like sequences were shuffled internally but transposable elements (TEs) were left untouched, or when only the TEs were shuffled internally, the α(ℓ) profile is almost identical to the original α(ℓ) profile of the natural sequence. This means that the TEs are the main contributors to the heterogeneity at intermediate scale, although introns and exons also make a contribution (see [19] for details).

### Phylogenetic distribution

**Figure 4:**
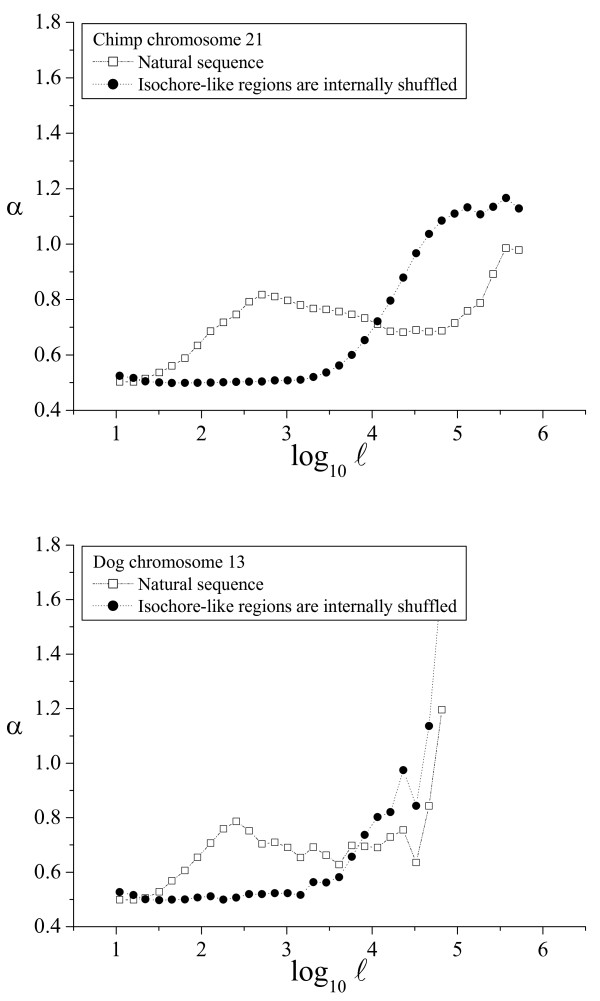
**α(ℓ) profiles in chimpanzee and dog**. The α(ℓ) profiles in chimpanzee and dog genomes. The profiles for the natural sequence of an example chromosome from each genome, and the corresponding artificial sequence obtained after the isochore-like regions were internally shuffled, are shown.

**Figure 5:**
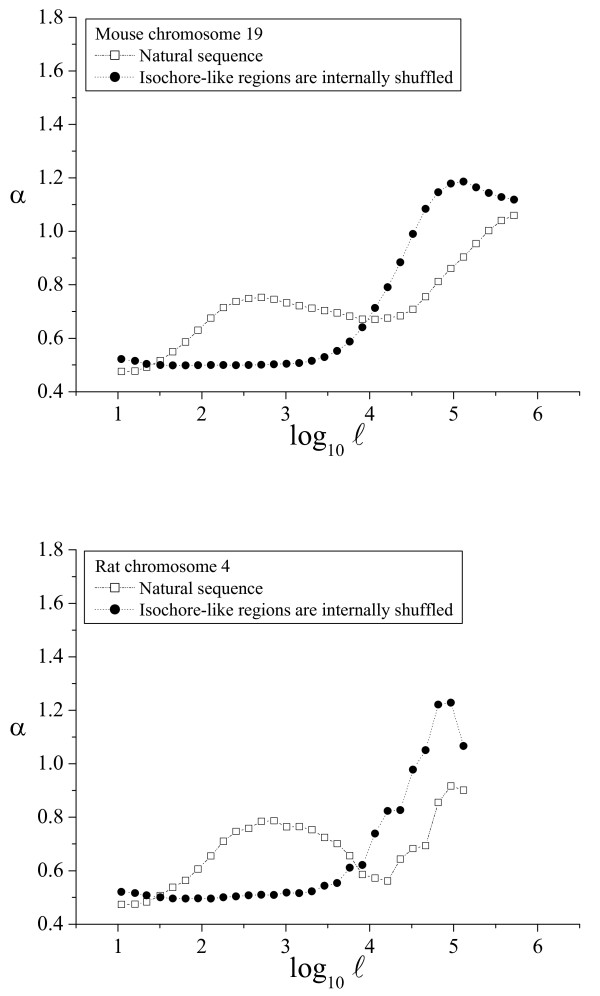
**α(ℓ) profiles in mouse and rat**. The α(ℓ) profiles in mouse and rat genomes. See the legend of Figure 4 for additional details.

**Figure 6:**
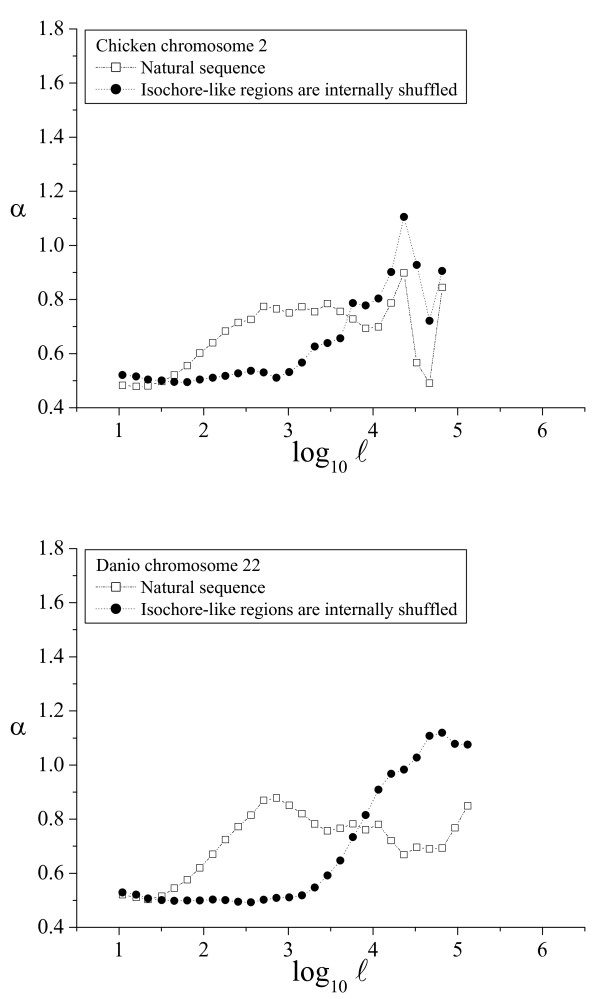
**α(ℓ) profiles in birds and fishes**. The α(ℓ) profiles in chicken and *Danio *genomes. See the legend of Figure 4 for additional details.

**Figure 7:**
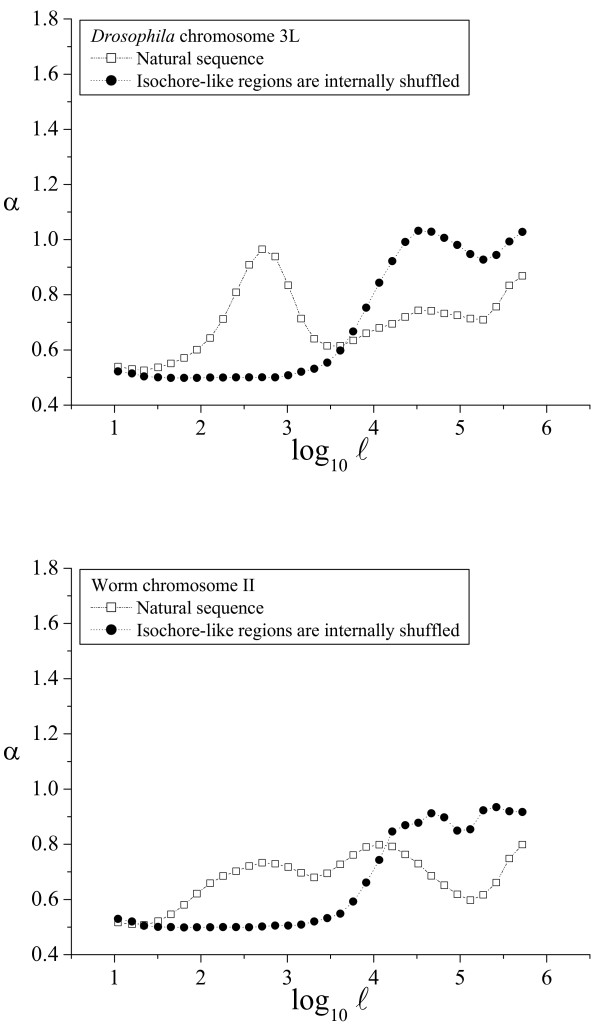
**α(ℓ) profiles in invertebrates**. The α(ℓ) profiles in *Drosophila *and worm genomes. See the legend of Figure 4 for additional details.

**Figure 8:**
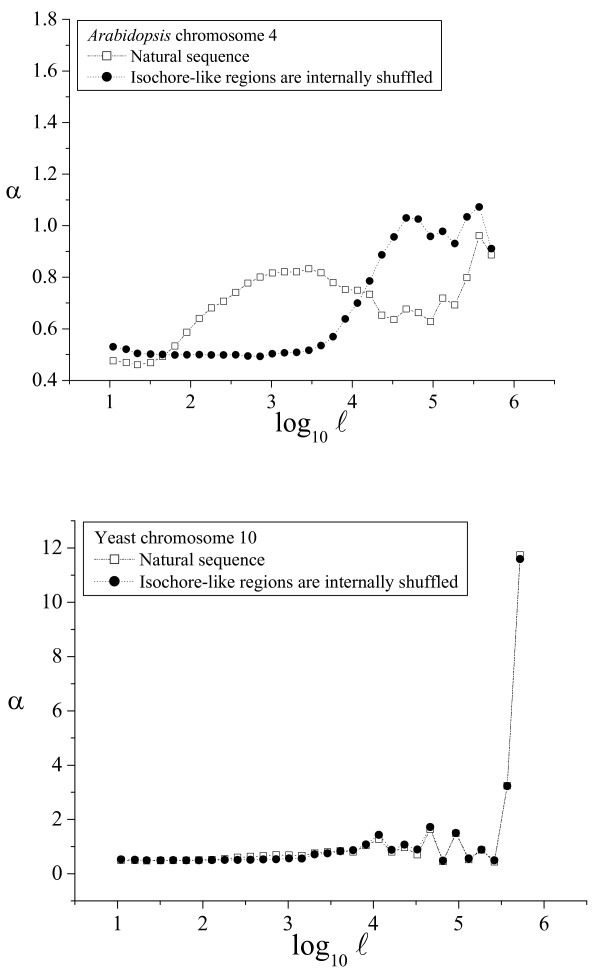
**α(ℓ) profiles in plants and yeasts**. The α(ℓ) profiles in *Arabidopsis *and yeast genomes. See the legend of Figure 4 for additional details.

As shown above, the α(ℓ) profile revealed as a simple and powerful method to determine the phylogenetic distribution of large-scale patchiness. Two α(ℓ) profiles have been already shown for the human genome in Figures 2 and 3. We have also plotted sample α(ℓ) profiles for good-quality chromosome sequences from chimpanzee and dog (Fig. 4), mouse and rat (Fig. 5), chicken and *Danio *(Fig. 6), *Drosophila *and *Caenorhabditis elegans *(Fig. 7) and *Arabidopsis thaliana *and *Saccharomyces cerevisiae *(Fig. 8). The numerical values to trace the α(ℓ) profiles for the remaining chromosomes with sequence contigs of good-quality and sufficient length from all these species are given in the Additional File 1.

The extension of the intermediate scale in the other vertebrate genomes is similar to that observed in the human genome, and also in the *Arabidopsis *genome, while in the invertebrates (*Drosophila *and *Caenorhabditis*) the intermediate scale reaches only to 3 kbp. Notably, in the yeast genome this intermediate scale does not exist at all. The beginning of the large scale in all the genomes analyzed ranged from 10 to 30 kbp, while the ending length often extended beyond 100 kbp, or even 1 Mb, depending mainly on the length of the available sequence contigs.

The phylogenetic distribution of compositional patchiness within vertebrates has been traditionally assessed by ultracentrifugation of bulk DNA [11,13,16], uncovering a clear isochore structure in mammals and birds [6,10] and also in some reptiles [16]. The α(ℓ) profiles used here reveal large-scale genome patchiness on a wider phylogenetic range, extending to invertebrates (*Drosophila *and *C. elegans*), plants (*A. thaliana*) and yeasts (*S. cerevisiae*).

Besides the profiles for the natural sequence, all these figures also include the profiles for the corresponding artificial sequence obtained by internally shuffling the isochore-like sequence regions, as predicted by IsoFinder [24]. In all the genomes analyzed, the large scale was practically unaffected (save the peak increase due to the homogenization at the small scales). Therefore, the large-scale patchiness observed in the α(ℓ) profiles in all these species can be attributed to the *in silico *determined, isochore-like regions.

## Discussion

Large-scale genome compositional heterogeneity has been traditionally revealed through analytical ultracentrifugation of bulk DNA in a large range of genomes (for recent reviews see [8,10]). However, at the sequence level, such demonstration has been hampered by the lack of a consistent, reliable, and widely applicable method. The α(ℓ) profiles used here prove to be an excellent tool for this task, allowing us to determine the extent of the different scales of genome compositional heterogeneity in a wide phylogenetic range: yeasts, plants, invertebrates, fishes, birds and mammals. Additionally, the selective shuffling experiments used here to specifically randomize selected genome segments, while not touching others, allow us to identify the *in silico *determined, isochore-like regions as the biological source for large-scale patchiness throughout the entire range of the species analyzed.

A clear limitation of our approach is that it depends critically on the availability of good-quality sequence contigs of sufficient length. For this reason, we have analyzed only the best genome assemblies from eleven eukaryotic species. However, as these species cover a wide phylogenetic spectrum (mammals, birds, fishes, invertebrates, plants and yeasts) the conclusions should be general.

In all these genomes, the large-scale patchiness revealed by α(ℓ) profiles can be associated with the isochore-like genome regions predicted by IsoFinder, thus emphasizing the reliability of this algorithm in predicting isochore-like regions at the sequence level.

## Conclusion

The analysis of the deviations in the power-law behavior of long-range correlations, through α(ℓ) profiles, allowed us to uncover large-scale genome structure in the eleven sequenced genomes for which sequence contigs of sufficient length and quality are available. Furthermore, through selective shuffling experiments, we were able to identify the computationally-determined, isochore-like structure of these genomes as the biological source for such large-scale patchiness.

## Methods

### Genomes analyzed

The following genome assemblies, all having sequence contigs of sufficient length, were downloaded from the UCSC Genome Bioinformatics site [25]: *Homo sapiens *(hg18), *Pan troglodytes *(panTro2), *Mus musculus *(mm8), *Rattus norvegicus *(rn4), *Canis familiaris *(canFam2), *Gallus gallus *(galGal3), *Danio rerio *(danRer4), *Drosophila melanogaster *(dm2), *Caenorhabditis elegans *(ce2), and *Saccharomyces cerevisiae *(sacCer1). The genome assembly for *Arabidopsis thaliana *(Arab) was downloaded from the NCBI FTP site [26]).

### Detrended Fluctuation Analysis (DFA) and deviations from perfect power-law behavior

The DFA method [2,27,28] is aimed to detect and quantify long-range correlations in numerical sequences, and therefore DNA sequences have to be mapped into numerical ones prior to the application of DFA. Thus, a DNA chain of length *N *is of the form *s*_1 _*s*_2 _... *s*_*N*_, and the *s*_*i *_values are obtained according to the strong-weak (SW) mapping rule: C or G → *s*_*i *_= 1, A or T → *s*_*i *_= 0. Note that this mapping rule is particularly appropriate to analyze genome-wide correlations, since it corresponds to the most fundamental partitioning of the four bases into their natural pairs in the double helix (A-T and G-C). As expected [29], when the purine-pyrymidine mapping rule was assayed, only structures at intermediate, but not at large, scale were detected (not shown).

To apply DFA, first we create the integrated series by accumulating the original one:

(1) $$y(j)=\sum_{i=1}^{j}[s_{i}-\bar{s}]$$

where $\bar{s}$ is the global mean:

(2) $$\bar{s}=\frac{1}{N}\sum_{i=1}^{N}s_{i}.$$

The integrated series *y(1) y(2) *... *y(N) *is divided into N_W _windows of equal length ℓ, and in each window we fit the integrated series by using a linear fit *y*_fit _(the local trend). Then, we detrend the integrated series by subtracting the local trend obtaining the detrended fluctuation function *Y*:

(3) *Y*(*i*) = *y*(*i*) - *y*_*fit*_(*i*)

Finally, we obtain the fluctuation function *F*(ℓ) in this way:

(4) $$F(\ell )=\sqrt{\frac{1}{N_{W}}\sum_{j=1}^{N_{W}}\left(\frac{1}{\ell }\sum_{k=1}^{\ell }Y_{j}^{2}(k)\right)}$$

where *Y*_*j*_*(k) *is the *k*-th point (*k = 1 *... ℓ) within the *j*-th window (*j = 1 ... N*_*W*_). Therefore, F(ℓ) accounts for the average fluctuation of the series around its local trend at scale ℓ.

Fractal long-range correlations appear when F(ℓ) scales in the form:

(5) F(ℓ) ∝ ℓ^*α*^

The exponent α quantifies the type and the strength of the correlations and can be determined by fitting *F*(ℓ) vs. ℓ to a straight line in a log-log plot, the slope being α (Fig. 1). If α = 0.5, there is no correlation and the sequence behaves as a random series (white noise), α < 0.5 indicates anti-correlations, and α > 0.5 positive correlations. However, this procedure can mask information present in the signal (see [19] for details) and we prefer to determine α as

α(ℓ) = d log (*F*(ℓ))/d log (ℓ)

In this way, deviations from power-law behavior lead to local variations of α which can be detected by plotting α(ℓ) vs. ℓ (Fig. 2). The DNA sequence shown in this figure, corresponding to the long contig of 92.1 Mb from human chromosome IV, clearly shows two main deviations from the power-law behavior, suggesting the presence of two main characteristic scales (the two major peaks in α(ℓ)) at intermediate and large ℓ values [19].

### Selective shuffling experiments

A shuffled sequence is a random permutation of the original DNA sequence and can be used to test hypotheses concerning any pattern uncovered in DNA sequences. Shuffling can affect the entire sequence or be restricted to only certain regions within a sequence in order to test the influence of such regions on the overall behavior of the one being observed. Here, we carried out selective-shuffling experiments by shuffling only those regions in the genome sequence corresponding to the isochore-like regions predicted by IsoFinder [24]. All the patterns within the isochore-like regions are destroyed by such shuffling, while those out of these regions remain intact. Selectivity in the shuffling process can go a step further when certain genome elements within the isochore-like regions (e.g. TEs) are preserved from shuffling, thereby also remaining intact while the rest of the region is randomized. We obtained both types of partially shuffled sequences in order to identify the biological source for the two length scales observed in the α(ℓ) profiles. The coordinates for isochore-like regions identified by IsoFinder on each chromosome sequence of the eleven genomes analyzed in this study are available at the Online Resource on Isochore Mapping [30].

## Authors' contributions

PC proposed the idea and, jointly with PBG, wrote the code to locate sequence contigs of sufficient length and compute the α(ℓ) profiles. MH wrote the code for selective-shuffling experiments. JLO designed the experimental setup, carried out the computations, wrote the original draft and edited coauthor's contributions. All the authors read and made contributions to the manuscript and approved the final version.

## Supplementary Material

Additional file 1 **Numerical values for the α(ℓ) profiles**. The numerical values to trace the α(ℓ) profiles for all the chromosomes with sequence contigs of sufficient lengths from the eleven species analyzed here are given (Excel file). Click here for file
